# Combination of α-Tomatine and Curcumin Inhibits Growth and Induces Apoptosis in Human Prostate Cancer Cells

**DOI:** 10.1371/journal.pone.0144293

**Published:** 2015-12-02

**Authors:** Huarong Huang, Xuan Chen, Dongli Li, Yan He, Yu Li, Zhiyun Du, Kun Zhang, Robert DiPaola, Susan Goodin, Xi Zheng

**Affiliations:** 1 Allan H. Conney Laboratory for Anticancer Research, Guangdong University of Technology, Guangzhou, P. R. China; 2 Department of Chemical and Environmental Engineering, Wuyi University, Jiangmen City, Guangdong Province, P.R. China; 3 Department of Environmental Engineering, Guangdong Industry Technical College, Guangzhou, P.R. China; 4 Cancer Institute of New Jersey, New Brunswick, New Jersey, United States of America; Rutgers, the State Univesity of New Jersey, UNITED STATES

## Abstract

α-Tomatine is a glycoalkaloid found in tomatoes and curcumin is a major yellow pigment of turmeric. In the present study, the combined effect of these two compounds on prostate cancer cells was studied. Treatment of different prostate cancer cells with curcumin or α-tomatine alone resulted in growth inhibition and apoptosis in a concentration-dependent manner. Combinations of α-tomatine and curcumin synergistically inhibited the growth and induced apoptosis in prostate cancer PC-3 cells. Effects of the α-tomatine and curcumin combination were associated with synergistic inhibition of NF-κB activity and a potent decrease in the expression of its downstream gene Bcl-2 in the cells. Moreover, strong decreases in the levels of phospho-Akt and phosphor-ERK1/2 were found in PC-3 cells treated with α-tomatine and curcumin in combination. In animal experiment, SCID mice with PC-3 xenograft tumors were treated with α-tomatine and curcumin. Combination of α-tomatine and curcumin more potently inhibited the growth of PC-3 tumors than either agent alone. Results from the present study indicate that α-tomatine in combination with curcumin may be an effective strategy for inhibiting the growth of prostate cancer.

## Introduction

Prostate cancer is one of the most common cancers in European and American males and has a high mortality rate [1]. Most patients demonstrate an initial response to hormonal manipulation but unfortunately the vast majority of patients progress to develop hormone-refractory disease. While newer anti-androgen treatment and chemotherapy options are available for patients with androgen-independent prostate cancer, these agents possess considerable toxicity and are only temporarily effective [2–5]. Therefore, novel and less toxic approaches for the treatment of prostate cancer would be of great benefit for patients.

**Fig 1:**
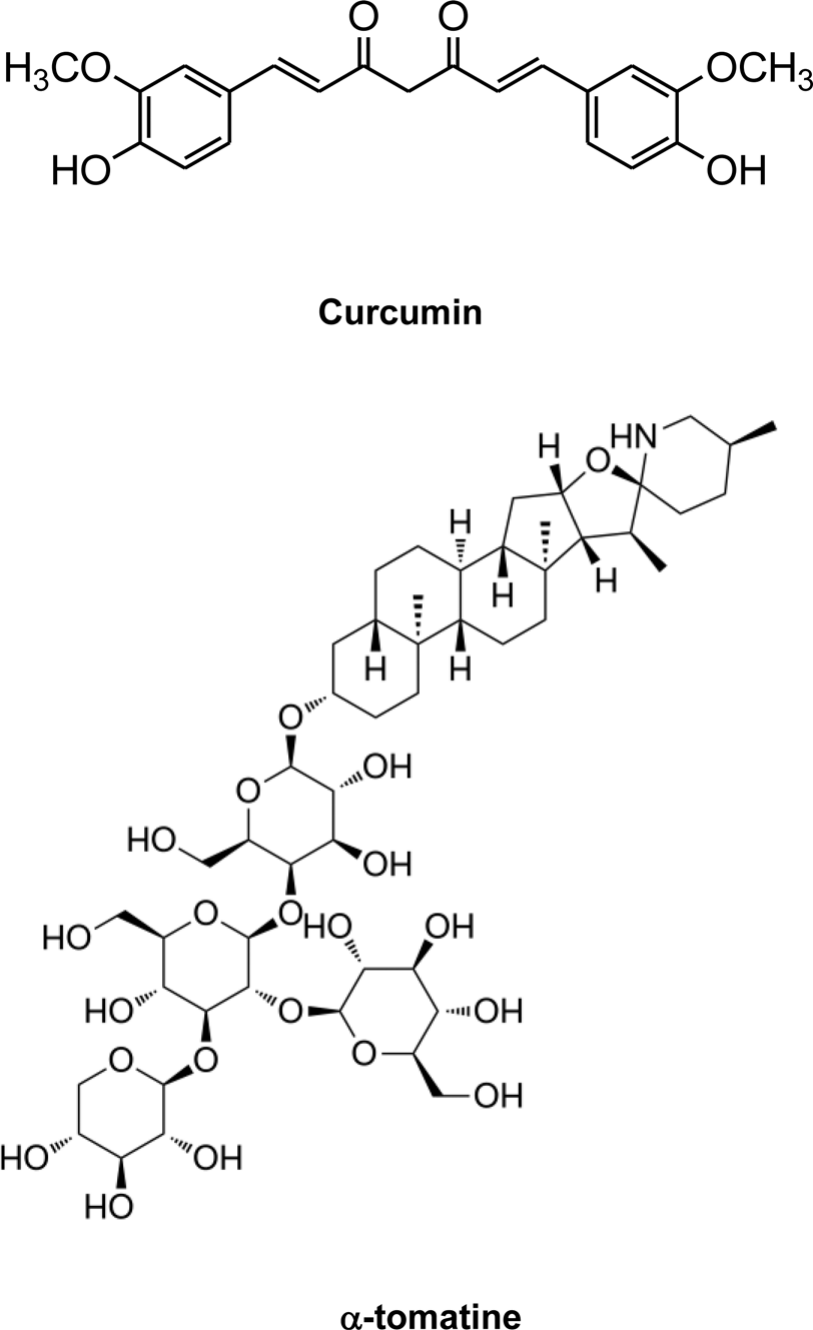
Structures of α-tomatine and curcumin.

Curcumin (Fig 1) is a major yellow pigment of turmeric *Curcuma longa* Linn. Turmeric is a common spice in Asian foods and has a long history of medicinal use in Asian countries. Curcumin has extensive biological and pharmacological functions including anticancer, anti-inflammatory, and antioxidant [6–8]. Curcumin has been evaluated in clinical trials for the treatment of liver disease, rheumatoid arthritis, infectious diseases and cancers [9, 10]. Despite its promising biological effects in preclinical studies, the clinical usefulness of curcumin is diminished by its poor bioavailability [11]. Although many curcumin analogues have been developed to improve the therapeutic efficacy, the bioavailability and toxic side effects of these compounds need further studies [12–18]. Combining curcumin with other anticancer agents is an effective strategy for improving its anticancer efficacy, and indeed previous studies have shown that combinations of curcumin with other anticancer agents have improved anticancer efficacies [19–21].

α-Tomatine (Fig 1) is a naturally occurred steroidal glycoalkaloid in tomatoes (*Lycopersicon* esculentum). Immature green tomatoes contain up to 500 mg α-tomatine/kg fresh fruit weight. The compound is partly degraded as the tomato ripens until at maturity levels in red tomatoes are about 5 mg/kg fresh fruit weight [22]. In plants, α-tomatine may provide defence against pathogenic fungi, bacteria and viruses [22]. The anticancer activities of α-tomatine and its mechanisms of action have been studied during recent years. In vitro studies demonstrated that α-tomatine inhibited the growth of different human cancer cells [23–25]. Recent studies also showed that α-tomatine inhibited the growth of mammary and prostate tumors in mice [26, 27]. A combination of α-tomatine and paclitaxel was found to synergistically enhance apoptosis of human prostate cancer cells [28].

There is increasing interest in using a combination of low doses of anticancer agents that differ in their modes of action rather than administering a single agent at a high dose. Combinations of anticancer agents that have different mechanisms of action may have synergistic effect on inhibiting the growth and inducing apoptosis in prostate cancer cells. Although the inhibitory effect of α-tomatine or curcumin on prostate cancer had been studied, no studies have examined the combined effect of these two agents on prostate cancer cells cultured in vitro and grown as xenograft tumors in vivo. Studies showed that the effects of curcumin and α-tomatine on cancer cells were associated with inhibition of NF-κB activation [7, 17, 24, 26]. We hypothesized that combination of low concentrations of curcumin and α-tomatine will synergistically inhibit NF-κB activation leading to strong growth inhibition and apoptosis induction in prostate cancer cells. The present study was therefore designed to explore the effect of α-tomatine in combination with curcumin at low concentrations on growth and apoptosis in human prostate cancer cells. Effects of α-tomatine and curcumin in combination on NF-κB and related molecular pathways were determined. Our study provided the first evidence that a combination of low concentrations of α-tomatine and curcumin strongly inhibited prostate cancer cells cultured in vitro and grown as xenograft tumors in immunodeficient mice.

## Materials and Methods

### Cell culture and reagents

RWPE-1, LNCaP, VCaP and PC-3 cells were obtained from the American Type Culture Collection (ATCC, Rockville, MD, USA). Curcumin, α-tomatine, propylene glycol, polysorbate 80, benzyl alcohol, ethanol and DMSO were from Sigma (St. Louis, MO). Matrigel was obtained from BD Biosciences (Bedford, MA). RPMI-1640 tissue culture medium, penicillin-streptomycin, L-glutamine and fetal bovine serum (FBS) were from Gibco (Grand Island, NY). RWPE-1 cells were maintained in serum-free keratinocyte medium (K-SFM; 17005–042) from Gibco (Grand Island, NY). LNCaP, VCaP and PC-3 cells were maintained in RPMI-1640 culture medium containing 10% FBS that was supplemented with penicillin (100 units/ml)-streptomycin (100 μg/ml) and L-glutamine (300 μg/ml). Cultured cells were grown at 37°C in a humidified atmosphere of 5% CO_2_ and were passaged twice a week.

### Determination of the number of viable cells

The number of viable cells after each treatment was determined using a hemacytometer under a light microscope (Nikon Optiphot, Japan). Cell viability was determined by the trypan blue exclusion assay, which was done by mixing 80 μl of cell suspension and 20 μl of 0.4% trypan blue solution for 2 min. Blue cells were counted as dead cells and the cells that did not absorb dye were counted as live cells.

### Assessment of apoptotic cells

Apoptosis was determined by morphological assessment in cells stained with propidium iodide (PI) [29]. Apoptotic cells were identified by classical morphological features including nuclear condensation, cell shrinkage, and formation of apoptotic bodies [29]. Apoptosis was also determined by the Annexin V/PI double staining assay using fluoresceinisothiocyanate (FITC)-labeled Annexin V/PI apoptosis detection kit (BD Bioscience, San Jose, CA) according to the manufacturer's instructions. Briefly, cells after treatment were collected, washed in cold phosphate-buffered saline (PBS) twice, stained with FITC-conjugated Annexin V and PI dyes. The externalization of phoshotidylserine and the permeability to PI were evaluated by FACS Calibur flowcytometer (BD Bioscience, San Jose, CA). Data from 10,000 gated events per sample were collected. Cells in early stages of apoptosis were positively stained with Annexin V. Cells in late apoptosis were positively stained with both Annexin V and PI.

### Western blot analysis

After treatment, the cell lysates were prepared as described earlier [30]. Proteins were subjected to sodium dodecyl sulfate polyacrylamide gel electrophoresis (SDS-PAGE) and transferred to nitrocellulose membrane. After blocking nonspecific binding sites with blocking buffer, the membrane was incubated overnight at 4°C with primary antibodies (#4051 for phosphor-Akt and #4376 for phosphor-Erk1/2, both from Cell Signaling Co., Beverly, MA; 05–729 for Bcl-2 from Millipore Co., Billerica, MA). The β-actin was used as a loading control. Following removal of the primary antibody, the membrane was washed three times with TBS (PBS containing 0.05% tween 20) buffer at room temperature and then incubated with fluorochrome-conjugated secondary antibody (Santa Cruz Biotechnology Inc., CA). The membrane was then washed with TBS three times. Final detection was done with Li-Cor Odyssey infrared imaging system (Li-Cor Biotechnology, Lincoln, NE).

### NF-κB-dependent reporter gene expression assay

NF-κB transcriptional activity was measured by the NF-κB-luciferase reporter gene expression assay. An NF-κB luciferase construct was stably transfected into PC-3 cells and a single stable clone, PC-3/N [17], was used in the present study. In brief, PC-3/N cells were treated with curcumin or α-tomatine alone or in combination for 24 h, and the NF-κB-luciferase activities were measured using the luciferase assay kits from Promega (Madison WI, USA). After treatments, the cells were washed with ice-cold phosphate buffered-saline (PBS) and harvested in 1 x reporter lysis buffer. After centrifugation, 10 μl aliquots of the supernatants were measured for luciferase activity by using a Luminometer from Turner Designs Instrument (Sunnyvale, CA, USA). The luciferase activity was normalized against known protein concentrations and expressed as percent of luciferase activity in the control cells, which were treated with DMSO solvent. The protein level was determined by Bio-Rad protein assay kits (Bio-Rad, Hercules, CA, USA) according to the manufacturer's instructions.

### Formation and growth of PC-3 tumors in immunodeficient mice

Male severe combined immunodeficient (SCID) mice (6–7 weeks old) were obtained from Taco Farms Inc (Germantown, NY). The animals were housed in sterile filter-capped microisolator cages and provided with sterilized food and water. Prostate cancer PC-3 cells (2 × 10^6^ cells/0.1 ml/mouse) suspended in 50% Matrigel (Collaborative Research, Bedford, MA) in RPMI 1640 medium were injected subcutaneously into the right flank of the mice. After about 4 week, mice with established xenograft tumors were injected with vehicle, α-tomatine (5 mg/kg), curcumin (5 mg/kg), or α-tomatine (5 mg/kg) + curcumin (5 mg/kg) once every three day for 30 days. Each group had 9 mice and all animals received same amount of vehicle (5 μl/g body weight) which consisted of propylene glycol, polysorbate 80, benzyl alcohol, ethanol and water (40: 0.5: 1: 10: 48.5). Tumor size (length x width) and body weight were measured every third day. At the end of the study, mice were sacrificed, tumors were excised, weighed and placed in phosphate-buffered formalin at room temperature for 48 h and then placed in ethanol for 48 h before preparing paraffin sections as previously described [31]. The animal study was conducted according to the recommendations in the Guide for the Care and Use of Laboratory Animals of the National Institute of Health. The protocol was approved by the Institutional Animal Care and Use Committee (IACUC) of Rutgers (RU02-001). This protocol approved our present animal study utilizing the SCID mouse xenograft model to determine the effects of naturally occurred compounds (α-tomatine and curcumin) on the growth of prostate tumors.

### Immunohistochemistry

Immunohistochemical staining of proliferating cell nuclear antigen (PCNA) was used to determine the proliferation of the PC-3 tumor cells. In brief, paraffin sections of tumor tissues were prepared and processed for immunohistochemical staining. The tumor sections were incubated with a primary PCNA antibody (MAB424, Millipore Corp. Billerica, MA, USA) for 1 h at room temperature. After washed with phosphate buffered saline (PBS), the sections were incubated with a biotinylated secondary antibody for 30 min followed by incubation with horseradish peroxidase conjugated-avidin solution for 30 min using the Elite ABC kit (PK-6100, Vector Laboratories, Burlingame, CA, USA). PCNA staining in tumor cells (brown color in nucleus) were examined under a microscope (Nikon Optiphot, Nikon, Tokyo, Japan). At least 1000 cells were counted for each section.

### Statistical analyses

The potential synergistic effect of α-tomatine or curcumin was assessed by the isobole method [32], using the equation Ac/Ae + Bc/Be = combination index (CI). Ac and Bc represent the concentration of drug A and drug B used in the combination, and Ae and Be represent the concentration of drug A and B that produced the same magnitude of effect when administered alone. If CI is <1, then the drugs are considered to act synergistically. If the CI is >1 or = 1, then the drugs act in an antagonistic or additive manner, respectively. The analysis of variance (ANOVA) model with Tukey-Kramer adjustment was used for the comparison of apoptosis in PC-3 cells among different treatment groups, and for the comparison of body weight, tumor size, tumor weight and tumor proliferation in animals among different treatment groups at the end of the experiment.

## Results

### α-Tomatine and curcumin inhibit the growth of prostate cancer cells

**Fig 2:**
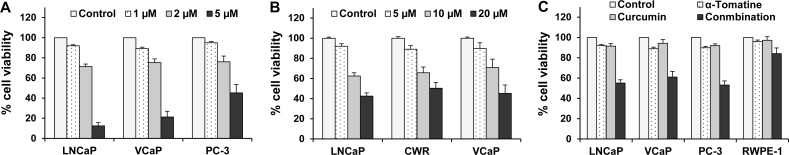
Effects of α-tomatine and curcumin alone or in combination on the growth of cultured prostate cancer cells. Human prostate cancer cells (LNCaP, VCaP and PC-3) and non-tumorigenic prostate epithelial cells (RWPE-1) were seeded at a density of 0.2 × 10^5^ cells/ml in 35 mm tissue culture dishes and incubated for 24 h. The cells were then treated with various concentrations of α-tomatine and curcumin alone or in combination for 72 h. Viable cells was determined by the trypan blue exclusion assay. (A) Percent cell viability in the various cell lines treated with α-tomatine. (B) Percent cell viability in the various cell lines treated with curcumin. (C) Percent cell viability in the various cell lines treated with α-tomatine (1 μM) and curcumin (5 μM) alone or in combination. Each value represents mean ± S.E from three separate experiments.

In initial studies, the effects of α-tomatine or curcumin alone in different prostate cancer cells were determined. Human prostate cancer LNCaP, VCaP (androgen-dependent) and PC-3 (androgen-independent) cells were treated with different concentrations of α-tomatine and curcumin for 72 h. Cell viability was determined by the trypan blue exclusion assay. As shown in Fig 2A, treatment of different prostate cancer cells with α-tomatine resulted in a concentration-dependent decrease in the number of viable cells. Treatment of the cells with curcumin also resulted in a concentration-dependent decrease in the number of viable cells (Fig 2B). The effects of curcumin and α-tomatine on cell growth were similar among the three prostate cancer cell lines tested. As shown in Fig 2C, α-tomatine and curcumin in combination had more potent inhibitory effect on LNCaP, VCaP and PC-3 cells. The combination index (CI) for IC_50_ was calculated as 0.69, 0.72 and 0.48 for LNCaP, VCaP and PC-3 cells, respectively. This result indicates that the combination of α-tomatine and curcumin synergistically inhibits the growth of cultured prostate cancer cells. Curcumin and α-tomatine alone or in combination had a small inhibitory effect on the growth of non-tumorigenic prostate epithelial RWPE-1 cells (Fig 2C).

### α-Tomatine and curcumin induce apoptosis in PC-3 cells

**Fig 3:**
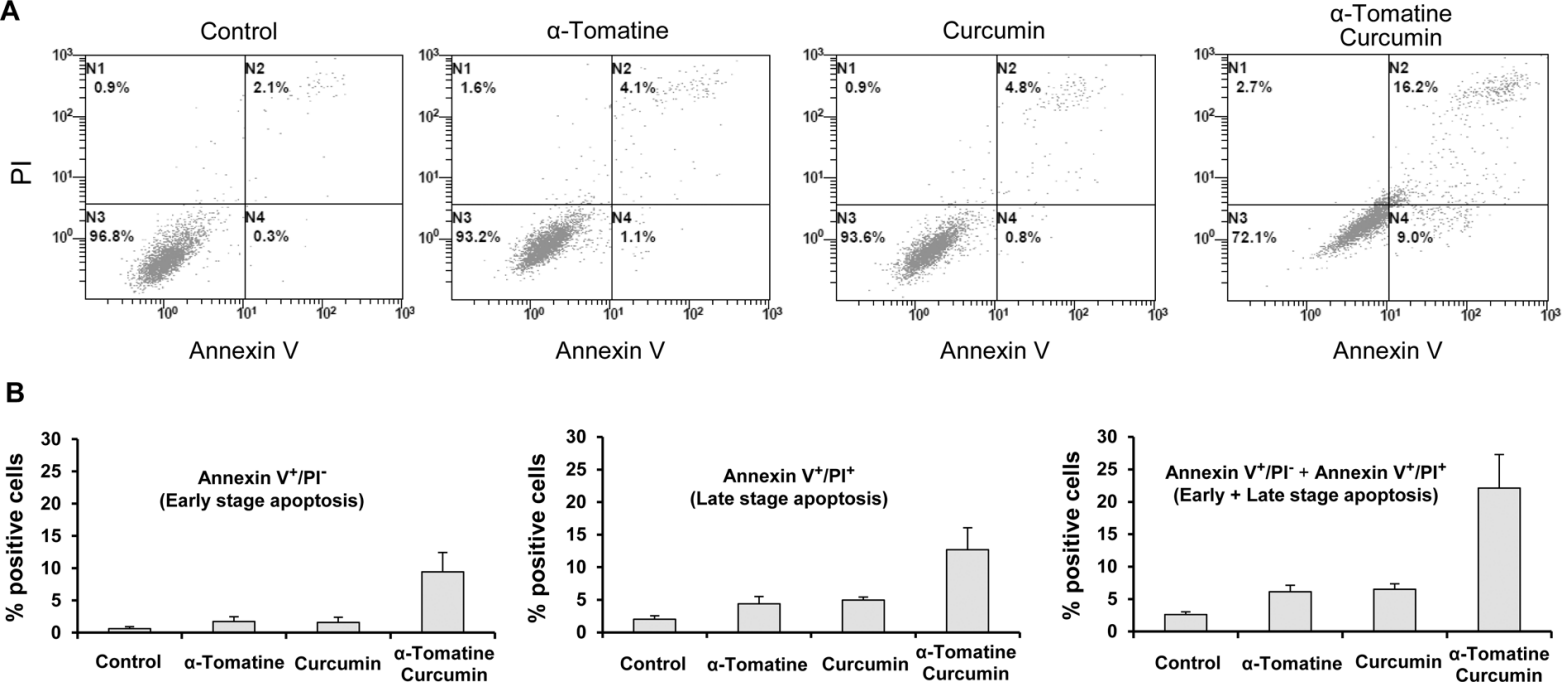
Effect of α-tomatine and curcumin on apoptosis of PC-3 cells. PC-3 cells were seeded at a density of 0.5 × 10^5^ cells/ml in 100 mm tissue culture dishes and incubated for 24 h. The cells were then treated with α-tomatine and curcumin alone or in combination for 48 h. Apoptosis was determined by the Annexin V/PI double staining assay using FITC-labeled Annexin V/PI apoptosis detection kit and flowcytometer analysis. (A) Representative result from flowcytometer analysis is shown. (B) Percentage of early stage apoptotic cells (left), late stage apoptotic cells (middle) and total apoptotic cells (right). Each value represents mean ± S.E from three separate experiments.

The effect of α-tomatine and curcumin on induction of apoptosis in prostate cancer cells were determined using morphological assessment and the Annexin V/PI double staining. As shown in Table 1, treatment of prostate cancer LNCaP, VCaP and PC-3 cells with α-tomatine or curcumin resulted in small increase in the number of apoptotic cells. Combinations of α-tomatine and curcumin had more potent stimulatory effect on apoptosis than either agent used alone. Curcumin and α-tomatine alone or in combination had a small to moderate effect on stimulation of apoptosis in non-tumorigenic prostate epithelial RWPE-1 cells (Table 1). Statistical analysis for synergy showed that α-tomatine and curcumin had a stronger synergistic effect on PC-3 cells (CI = 0.77) than on LNCaP (CI = 0.90) and VCaP (CI = 0.96) cells. Since the combination of α-tomatine and curcumin had a stronger synergistic effect on apoptosis induction in the androgen-independent PC-3 cells, we chose the PC-3 line for further *in vitro* and *in vivo* studies. Induction of apoptosis in PC-3 cells treated with α-tomatine and curcumin was also determined in experiments using the Annexin V/PI double staining. In these analyses, cells in the early stages of apoptosis were stained positively with Annexin V, whereas cells in late stages of apoptosis were stained positively with both Annexin V and PI. Fig 3A shows a representative result from flowcytometer analysis. As shown in Fig 3B, α-tomatine (1 μM) or curcumin (5 μM) had small to moderate effect on stimulation of apoptosis, and the combination of the two agents caused a substantial increase in apoptosis. Statistical analysis using ANOVA with the Tukey-Krama multiple comparison test showed that the percentage of apoptotic cells in the combination group was significantly higher than that in the α-tomatine-treated group (*p* < 0.001) and that in curcumin-treated group (*p* <0.001).

**Table 1:** Effect of α-tomatine and curcumin on apoptosis of cultured prostate cancer cells.

	% apoptotic cells			
Treatment	LNCaP	VCaP	PC-3	RWPE-1
Control	1.6 ± 0.4	1.3 ± 0.3	1.1 ± 0.2	0.9 ± 0.2
α-T (1 uM)	4.6 ± 0.6	5.3 ± 0.5	6.6 ± 0.7	1.5 ± 0.3
Cur (5 uM)	6.9 ± 0.7	6.2 ± 0.6	5.8 ± 0.4	2.9 ± 0.2
Cur (10 uM)	12.1 ± 1.0	9.8 ± 0.8	11.4 ± 1.1	5.2 ± 0.7
α-T (1 uM) + Cur (5 uM)	21.6 ± 2.3	19.5 ± 2.9	24.6 ± 3.1	7.8 ± 0.4
α-T (1 uM) + Cur (10 uM)	38.5 ± 4.1	36.8 ± 3.7	40.7 ± 5.0	13.9 ± 1.1

LNCaP, VCaP, PC-3 and RWPE-1 cells were seeded at a density of 0.2 × 10^5^ cells/ml and incubated for 24 h. The cells were then treated with α-tomatine (α-T; 1 μM) or curcumin (Cur; 5 or 10 μM) alone or in combination for 48 h. Apoptosis was determined by morphological assessment. Each value is the mean ± S.E from three experiments.

### Inhibitory effect of α-tomatine and curcumin on NF-κB and its down-stream target Bcl-2

**Fig 4:**
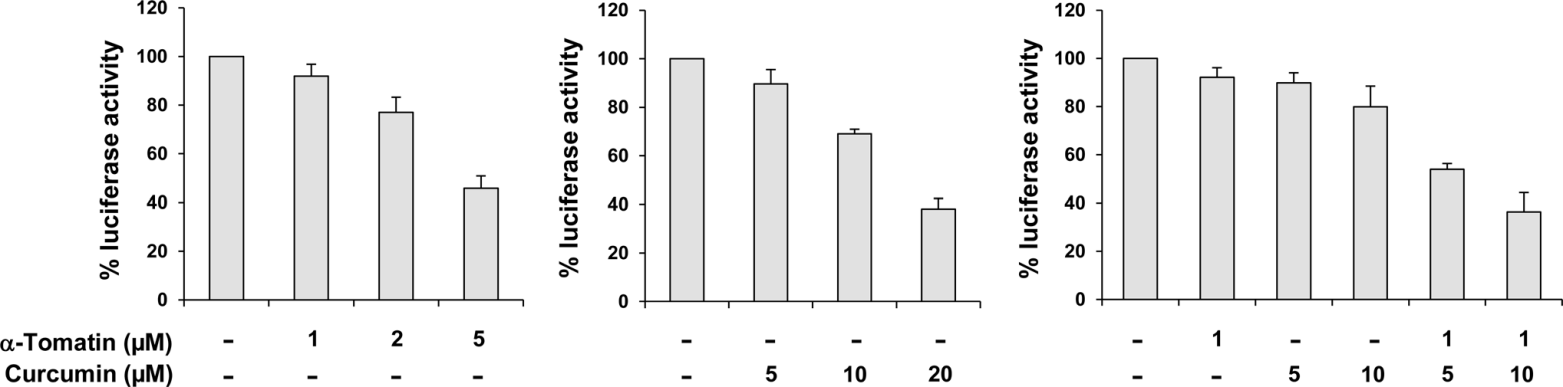
Inhibitory effect of α-tomatine and curcumin alone or in combination on NF-κB activation in PC-3 cells. PC-3/N cells were seeded at a density of 0.2×10^5^ cells/ml of medium in 12-well plates and incubated for 24 h. The cells were then treated with α-tomatine alone or in combination with curcumin for 24 h. The NF-κB transcriptional activity was measured by a luciferase activity assay. Each value represents mean ± S.E from three separate experiments.

**Fig 5:**
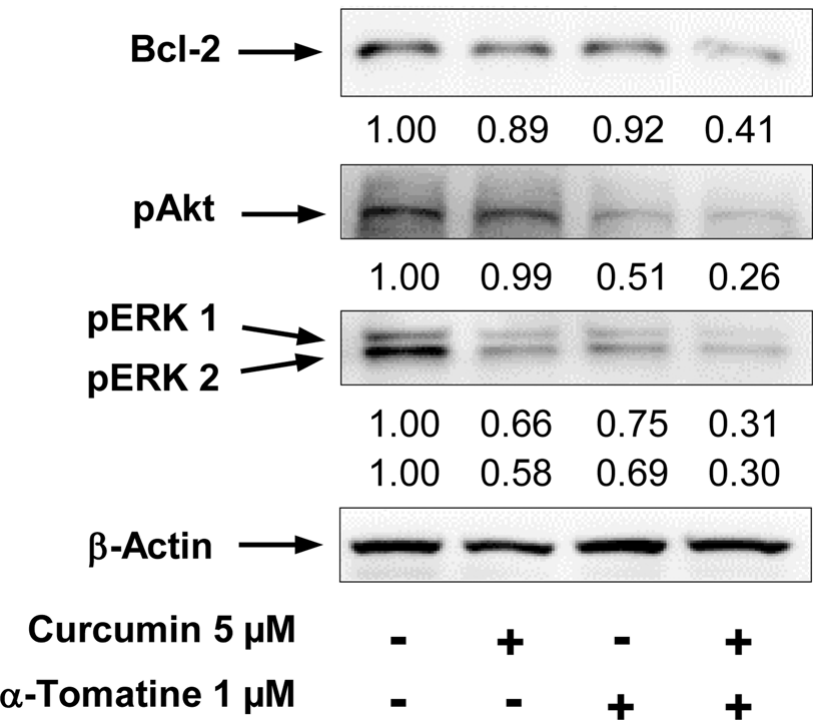
Effect of α-tomatine and curcumin on the level of Bcl-2, phospho-Akt and phospho-ERK1/2 in PC-3 cells. The cells were seeded at a density of 1×10^5^ cells/ml of medium in 100 mm culture dishes and incubated for 24 h. The cells were then treated with α-tomatine or curcumin alone and in combination for 24 h (for analysis of phosphor-Akt and phosphor-ERK1/2) and 48 h (for analysis of Bcl-2). The levels of Bcl-2, phospho-Akt and phospho-ERK1/2 were determined by the Western blot analysis. The band density was measured and normalized for actin.

A luciferase reporter gene expression assay was used to determine the effect of α-tomatine and curcumin on activation of NF-κB. PC-3/N is a cell line derived from stable transfection of PC-3 cells with a NF-κB luciferase construct [17]. PC-3/N cells were treated with α-tomatine and curcumin alone or in combination for 24 h. Treatment of PC-3/N cells with α-tomatine (1–5 μM) or curcumin (5–20 μM) alone resulted in decreases in luciferase activity in concentration-dependent manner (Fig 4). Lower concentrations of α-tomatine (1 μM) or curcumin (5 or 10 μM) had small to moderate inhibitory effects on luciferase activity, and the combinations of α-tomatine (1 μM) and curcumin (5 or 10 μM) had much stronger effects than either agent alone (Fig 4). Statistical analysis for synergy showed that α-tomatine and curcumin in combination had a synergistic effect on decreasing the NF-κB luciferase activity (CI = 0.56). The effect of α-tomatine and/or curcumin on the level of anti-apoptotic protein Bcl-2 which is a down-stream target of NF-κB was determined by the Western blot analysis. Treatment with α-tomatine or curcumin alone had little or no effect on the level of Bcl-2 while the combination had a strong inhibitory effect on the expression of this protein (Fig 5). The band density measurement showed that the level of Bcl-2 relative to control (1.00) was 0.89 in cells treated with curcumin, 0.92 in cells treated with α-tomatine and 0.41 in cells treated with the combination of α-tomatine or curcumin (Fig 5).

### Effects of α-tomatine and curcumin on the level of phospho-Akt and phospho-ERK1/2

The level of activated ERK1/2 and Akt in PC-3 cells was evaluated by Western blot analysis using anti phospho-ERK1/2 and phospho-Akt antibodies. Treatment of PC-3 cells with α-tomatine (1 μM) resulted in a moderate to strong decrease in the level of phospho-Akt while curcumin (5 μM) alone had little or no effect (Fig 5). A combination of α-tomatine and curcumin had a potent effect on decreasing the level of phospho-Akt. Band density measurement showed that the level of phospho-Akt relative to control (1.00) was 0.99 in cells treated with curcumin, 0.51 in cells treated with α-tomatine and 0.26 in cells treated with the combination of α-tomatine and curcumin (Fig 5). Treatment of PC-3 cells with α-tomatine (1 μM) or curcumin (5 μM) alone resulted in small to moderate decrease in the level of phospho-ERK1/2, and the combination of α-tomatine (1 μM) and curcumin (5 μM) caused a stronger decrease in the level of phospho-ERK1/2 than either agent alone (Fig 5). The level of phospho-ERK1 relative to control (1.00) as determined by band density measurement was 0.66 in cells treated with curcumin, 0.75 in cells treated with α-tomatine and 0.31 in cells treated with the combination of α-tomatine and curcumin (Fig 5). The level of phospho-ERK2 relative to control (1.00) was 0.58 in cells treated with curcumin, 0.69 in cells treated with α-tomatine and 0.30 in cells treated with the combination of α-tomatine and curcumin (Fig 5).

### Effects of α-tomatine and curcumin on the growth of PC-3 tumors in SCID mice

**Fig 6:**
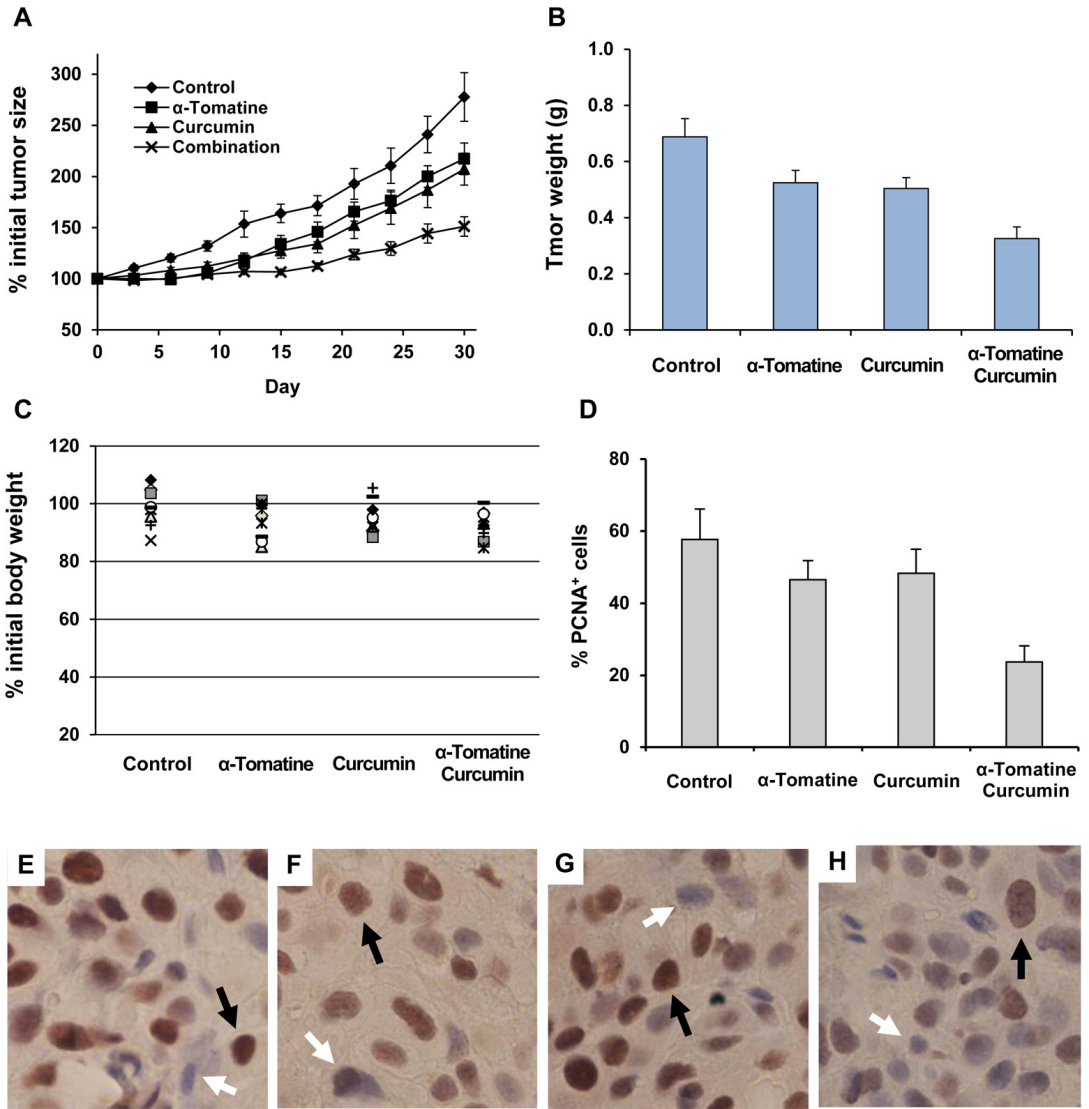
Effects of α-tomatine and curcumin alone or in combination on the growth of PC-3 xenograft tumors and body weight of SCID mice. Male SCID mice were injected subcutaneously with PC-3 cells (2 × 10^6^ cells/0.1 ml) suspended in 50% Matrigel in RPMI medium. After about 4 weeks, mice with PC-3 xenograft tumors (0.6–1.0 cm wide and 0.6–1.0 cm long) were i.p injected with vehicle, α-tomatine (5 mg/kg body weight), curcumin (5 mg/kg body weight), and a combination of α-tomatine (5 mg/kg body weight) and curcumin (5 mg/kg body weight) once every three day for 30 days. Immunohistochemical staining of PCNA was done to determine the effect of the various treatments on tumor cell proliferation. (A) Tumor size was expressed as percent of initial tumor size. (B) The weight (g) of each tumor was measured at the end of the experiment in mice after sacrifice. (C) Body weight was expressed as percent of initial body weight. (D) Percentage of PCNA positive cells in tumors from animals treated with vehicle, α-tomatine, curcumin or the combination of α-tomatine and curcumin. Representative micrographs of PCNA immunohistochemical staining in tumors from the control group (E), the α-tomatine-treated group (F), the curcumin-treated group (G) and the combination-treated group (H) are shown. Black arrows indicate positive PCNA staining and white arrows indicate negative PCNA staining.

SCID mice bearing PC-3 xenograft tumors were treated with i.p injections with vehicle (5 μl/g body weight), α-tomatine (5 mg/kg), curcumin (5 mg/kg), or α-tomatine (5 mg/kg) + curcumin (5 mg/kg) three times a week for 30 days. As shown in Fig 6A, treatment with α-tomatine or curcumin alone had a moderate inhibitory effect on the growth of PC-3 tumors while the combination had a potent effect on inhibiting the growth of the tumors. The mean ± S.E. for percent initial tumor size at the end of the experiment was 277.6 ± 18.6 for the control group, 217.3 ± 10.5 for the α-tomatine-treated group, 207.3 ± 15.6 for the curcumin-treated group, 151.2 ± 9.6 for the combination-treated group. Statistical analysis using ANOVA with the Tukey-Krama multiple comparison test showed statistically significant differences in the average tumor size between the control group and the α-tomatine-treated group (*p* < 0.05), between the control group and the curcumin-treated group (*p* < 0.01), and between the control group and the combination-treated group (*p* < 0.001). The average tumor size in the combination group was significantly smaller than that in the α-tomatine-treated group (*p* < 0.05) and that in curcumin-treated group (*p* <0.05).

The weight of each tumor was also measured in each mouse at the end of the experiment after sacrifice. Tumor weight of individual mice in each treatment group is shown in Fig 6B. The mean ± S.E. for tumor weight (g) was 0.69 ± 0.05 for the control group, 0.52 ± 0.04 for the α-tomatine-treated group, 0.50 ± 0.04 for the curcumin-treated group, 0.33 ± 0.04 for the combination-treated group. Statistical analysis using ANOVA with the Tukey-Krama multiple comparison test showed that the average tumor weight in the combination group was significantly lower than that in the α-tomatine-treated group (*p* < 0.05) and that in curcumin-treated group (*p* <0.05). An good relationship between tumor size (as measured in live animals before sacrifice) and tumor weight (measure after sacrifice) in individual mice was obtained (*r* = 0.804). The effect of the various treatments on body weight is shown in Fig 6C. Statistical analysis using ANOVA with the Tukey-Krama multiple comparison test showed that the differences in the percent of initial body weight between the control group and any of the treatment group were not statistically significant (*p* > 0.05).

### Effects of α-tomatine and curcumin on the proliferation of PC-3 tumors

The effects of α-tomatine and curcumin on the proliferation of PC-3 tumors were investigated by determining the expression of proliferating cell nuclear antigen (PCNA) in tumor cells. Paraffin sections of PC-3 tumors were stained with PCNA antibody. As shown in Fig 6D, treatment of the mice with α-tomatine or curcumin alone decreased the number of PCNA positive cells in the tumors. Combined treatment with α-tomatine and curcumin had a more potent effect on decreasing the number of PCNA positive cells than either agent used alone (Fig 6D). The differences in the number of PCNA positive cells were statistically significant between the combination-treated group and the α-tomatine-treated group (*p* <0.01), and between the combination-treated group and the curcumin-treated group (*p* <0.01). A good correlation between PCNA positive cells and tumor weight in individual mice was found (*r* = 0.72).

## Discussion

Although earlier studies showed that α-tomatine or curcumin inhibited prostate cancer cells [17, 26, 28, 33, 34], the effects and mechanisms of these two agents in combination on growth and apoptosis of prostate cancer cells *in vitro* and *in vivo* have not been reported. In the present study, we tested our hypothesis that low concentrations of curcumin and α-tomatine in combination will synergistically inhibit NF-κB activation leading to strong growth inhibition and apoptosis induction in prostate cancer cells. Our study demonstrated that α-tomatine and curcumin in combination synergistically inhibited the growth and induced apoptosis in prostate cancer PC-3 cells, and these effects were associated with synergy of the two compounds on decreasing NF-κB activity. Previous studies showed that a higher concentration of curcumin was required to inhibit the growth of prostate cancer cells (IC_50_ ≈ 20 μM) [16, 35]. However, the bioavailability of curcumin is low [11]. An effective strategy is to combine a low concentration of curcumin with other anticancer agent. Combinations of low doses of anticancer agents that work by different mechanisms may be more effective with less toxicity than individual compounds at higher dose levels. In our study, we found that a low concentration of curcumin (5 μM) in combination with a low concentration of α-tomatine (1 μM) synergistically inhibited the growth of cultured prostate cancer cells. In addition, this combination strongly inhibited the growth of PC-3 xenograft tumors in SCID mice. To the best of our knowledge, this is the first report indicating a strong combined effect of low concentrations of α-tomatine and curcumin on prostate cancer cells.

The transcription factor NF-κB is an important regulator for cell growth and survival in a variety of cells including prostate cancer cells [36–38]. NF-κB has been shown to be constitutively activated in invasive prostate cancer[39–41]. Activation of NF-κB is related to prostate cancer progression due to transcriptional regulation of its responsive genes [41]. In addition, NF-κB activation predicts a high risk of relapse in patients with localized disease [40, 42]. Therefore, NF-κB may serve as a therapeutic target for the treatment of prostate cancer. Curcumin at higher concentrations (20–50 μM) was shown to inhibit activation of NF-κB [17, 43]. However, a high concentration of curcumin is difficult to achieve in the blood. We found in the present study that a low concentration of curcumin (5 μM) combined with a low concentration of α-tomatine (1 μM) synergistically inhibited the NF-κB transcriptional activity in PC-3 cells. Synergistic inhibition of NF-κB activity may lead to a strong down regulation of its anti-apoptotic genes. Indeed, our present study showed that the level of Bcl-2 was strongly decreased in PC-3 cells treated with α-tomatine in combination with curcumin. Bcl-2 is a downstream target of NF-κB and is known to have anti-apoptotic activity [44]. Results of the present study suggest that a robust inhibition of NF-κB mediated anti-apoptotic pathway may contribute to the synergistic effect of the combination of α-tomatine and curcumin on induction of apoptosis in PC-3 cells.

Earlier studies have implicated activation of the Akt signaling pathway for the survival of prostate cancer cells treated with androgen ablation therapy [45, 46]. Both α-tomatine and curcumin were shown to inhibit Akt [24, 28, 47, 48]. In the present study, we found that α-tomatine alone decreased the level of phospho-Akt while curcumin alone at a low dose (5 μM) had very little effect on the level of phospho-Akt. The combination of low concentrations of α-tomatine and curcumin strongly decreased the level of phospho-Akt. Another signaling pathway that is associated with prostate cancer growth and progression is the mitogen activation protein kinase (MAPK). Constitutive activation of ERK1/2, a member of the MAPK family, has been observed in prostate cancer [49–50]. In the present study, we found that the combination of α-tomatine and curcumin had a potent effect on decreasing the level of ERK1/2 in the cells. Further studies are needed to determine whether the strong combined effect of α-tomatine and curcumin on Akt and ERK1 contribute to the synergistic effect of the two compounds on inhibiting NF-κB. Although our preliminary study showed that α-tomatine in combination with curcumin inhibited the AR activity in VCaP cells as determined by the luciferase reporter assay, the strong effect of this combination on androgen-independent PC-3 cells found in the present study indicates that AR independent mechanisms were involved. Future studies are needed to determine the effect of α-tomatine combined with curcumin on AR signaling in androgen-dependent prostate cancer cells.

In the animal experiment, a strong inhibitory effect of α-tomatine in combination with curcumin on the growth of PC-3 xenograft tumors in SCID mice was observed. Curcumin or α-tomatine alone had a moderate inhibitory effect on the growth of PC-3 tumors. A combination of the two agents more potently inhibited the growth of PC-3 tumors than either agent used alone. The doses for α-tomatine and curcumin used in the present study were chosen based on the effective doses of these two agents in previous publications [26, 28, 51, 52]. Lower doses of α-tomatine or curcumin alone only had small to moderate effect that will allow us to determine the strong combined effect of these two agents. In the present study, administration of α-tomatine (5 mg/kg body weight) and curcumin (5 mg/kg body weight) alone or in combination by i.p injection did not cause body weight loss in the animals. In addition, no abnormalities were found in the major organs at the end of the experiment indicating that α-tomatine and curcumin at the doses used in the present study were not toxic to the animals.

In summary, our present study demonstrated that α-tomatine and curcumin in combination strongly inhibited the growth and induced apoptosis in human prostate cancer cells. The effects of α-tomatine and curcumin on growth inhibition and apoptosis in prostate cancer cells were associated with inhibition of NF-κB activation and decreased levels of Bcl-2, phospho-Akt and phospho-ERK1/2. In addition, treatment of SCID mice with α-tomatine + curcumin strongly inhibited the growth of xenograft PC-3 tumors. The combination of α-tomatine and curcumin may be an effective approach for inhibiting the growth of prostate cancer.
